# A Fully-Automatic Multiparametric Radiomics Model: Towards Reproducible and Prognostic Imaging Signature for Prediction of Overall Survival in Glioblastoma Multiforme

**DOI:** 10.1038/s41598-017-14753-7

**Published:** 2017-10-30

**Authors:** Qihua Li, Hongmin Bai, Yinsheng Chen, Qiuchang Sun, Lei Liu, Sijie Zhou, Guoliang Wang, Chaofeng Liang, Zhi-Cheng Li

**Affiliations:** 1 Institute of Biomedical and Health Engineering, Shenzhen Institutes of Advanced Technology, Chinese Academy of Sciences, Shenzhen, China; 2 Department of Neurosurgery, Guangzhou General Hospital of Guangzhou Military Command, Guangzhou, China; 3 Department of Neurosurgery/Neuro-oncology, Sun Yat-sen University Cancer Center, State Key Laboratory of Oncology in South China, Collaborative Innovation Center for Cancer Medicine, Guangzhou, China; 4 Department of Neurosurgery, The 3rd Affiliated Hospital of Sun Yat-sen University, Guangzhou, China

## Abstract

In fully-automatic radiomics model for predicting overall survival (OS) of glioblastoma multiforme (GBM) patients, the effect of image standardization parameters such as voxel size, quantization method and gray level on model reproducibility and prognostic performance are still unclear. In this study, 45792 multiregional radiomics features were automatically extracted from multi-modality MR images with different voxel sizes, quantization methods, and gray levels. The feature reproducibility and prognostic performance were assessed. Multiparametric and fixed-parameter radiomics signatures were constructed based on a training cohort (60 patients). In an independent validation cohort (32 patients), the multiparametric signature achieved better performance for OS prediction (C-Index = 0.705, 95% CI: 0.672, 0.738) and significant stratification of patients into high- and low-risk groups (P = 0.0040, HR = 3.29, 95% CI: 1.40, 7.70), which outperformed the fixed-parameter signatures and conventional factors such as age, Karnofsky Performance Score and tumor volume. This study demonstrated that voxel size, quantization method and gray level had influence on reproducibility and prognosis of radiomics features for GBM OS prediction. An automatic method to determine the optimal parameter settings was provided. It indicated that multiparametric radiomics signature had the potential of offering better prognostic performance than fixed-parameter signatures.

## Introduction

Glioblastoma multiforme (GBM), the most frequent malignant primary brain tumor in adults, remains a big therapeutic challenge. The median survival is only 12–14 months^1^. The poor prognosis is mainly due to the intra-tumor heterogeneity. The heterogeneity poses clear challenge to target therapies based on invasive biopsy-based genomics. This challenge can be addressed by medical imaging which is non-invasive, repeatable and provides information of the entire tumor^2^.

Radiomics is an emerging imaging-based technique explicitly designed to extract high-throughput quantitative imaging features from standard of care images, convert the features into minable data, and build predictive or prognostic models linking image features to tumor phenotype^3^. Radiomics provides a tool for comprehensive quantification and visualization of intra-tumor heterogeneity at the radiological level. It aims to improve the decision-support for cancer therapy with a low-cost and repeatable solution. Recently, several radiomics studies have been conducted in different cancer sites such as lung^4^, head & neck^5^, colorectal^6,7^, and glioma^8^. For GBM, several imaging biomakers have been proposed via radiomics approaches to predict the survival^9^, treatment response^8^ and molecular characteristics^10^.

Despite the promising results, current radiomics studies still face challenges. One critical issue is the reproducibility, preventing radiomics techniques from practical use. The factors affecting the reproducibility include image acquisition^11,12^, intra- and inter-rater variations when employing manual or semi-automatic segmentations^5^, and image standardization process^13^. In clinical practice, multi-modality MRI data are acquired from a variety of machines with different protocols, hence image standardization should be performed to normalize the resolution and intensity prior to radiomics analysis. For radiomics models that employ fully-automatic segmentation, the human factors can be eliminated while the image standardization parameters play a major role among factors affecting the reproducibility. On the other hand, the image standardization parameters may also affect the prognostic performance. Several studies investigated the effects of CT or PET acquisition parameters on reproducibility and prognostic power of radiomics models, in which manual segmentations were employed^12,14^. In fully-automatic radiomics model the effects of the image standardization parameters such as voxel size, quantization method and gray level on model reproducibility and prognostic performance are still unclear. To our best knowledge, most current radiomics models extracted image features at fixed image standardization parameters. Because for different image features the optimal voxel sizes, quantization methods and gray levels may vary, the potential of radiomics models with fixed image standardization parameters may be limited.

In this paper, we proposed a fully-automatic multiparametric radiomics model for prediction of overall survival (OS) in GBM patients. The effects of image standardization parameters such as voxel size, quantization method and gray level on feature reproducibility and prognostic performance were investigated. The prognostic performance of multiparametric radiomics signature was compared with fixed-parametric signatures and conventional prognostic factors. The aim was to automatically construct a reproducible and prognostic multi-feature radiomics signature for GBM OS prediction, and to determine the optimal voxel size, quantization method and gray level for such a model.

## Methods

### Patients

The patient cohorts involved in this retrospective study consisted of two groups: a training cohort and an independent validation cohort. The training cohort consisted of 60 patients from the Cancer Genome Archive (TCGA) database. Another 32 patients comprising 11 from Sun Yat-Sen University Cancer Center (SYSUCC), 10 from Guangzhou General Hospital of Guangzhou Military Command, and 11 from The 3rd Affiliated Hospital of Sun Yat-sen University were used for independent validation. The imaging procedure, data processing and experiment design were approved by Sun Yat-Sen University Cancer Center Ethics Committee, Guangzhou General Hospital of Guangzhou Military Command Ethics Committee, and The 3rd Affiliated Hospital of Sun Yat-sen University Ethics Committee. All methods were performed in accordance with the relevant guidelines and regulations. Because the patient data in TCGA was deidentified, institutional review board approval for the training data was not required. For the validation data, informed consent was obtained from all subjects.

The demographic and clinical characteristics of the patients is listed in Table 1. The inclusion criteria were: (1) newly diagnosed and treatment-naive GBM, (2) availability of overall survival information, (3) availability of pre-treatment MR imaging including T1-weighted, T1-weighted Gadolinium contrast-enhanced, T2-weighted, and T2-weighted FLAIR images (T1, T1C, T2, and FLAIR), (4) MR images with diagnostic image quality. The exclusion criteria were patients with a history of treatment and patients missing survival information. The MRI data of the training cohort was obtained from the Cancer Imaging Archive (TCIA) that include imaging data corresponding to TCIA patients. Overall survival is calculated from the initial pathologic diagnosis date to death or censored point if still alive.

**Table 1:** Demographic and Clinical Characteristics of Patients in the Training and Validation Data Set.

Parameters	Training Data Set	Validation Data Set
No. of Patients	60	32
Sex		
Male	35	16
Female	25	16
Age(y)		
Range	10–81	31–84
Median	57.5	57.0
Mean	54.9	56.4
KPS		
Mean	80.9	82.6
OS(d)		
Range	30–1561	36–1642
Median	427	442
Mean	488	545

### MR Imaging

All local MR images were acquired with 3.0-T MR imaging systems (Magnetom Verio or Trio TIM, Siemens Healthcare, Erlangen, Germany) using standard head coils. T1 and T1C images were acquired with magnetization prepared rapid gradient echo sequence with: repetition time msec/echo time msec, 210-1710/4-20; section thickness, 2.0–5.0 mm; intra-section spacing, 0.37–1.02 mm. T2 images were obtained with: repetition time msec/echo time msec, 6000-10000/95-140; section thickness, 3.0–5.0 mm; intra-section spacing, 0.39–1.00 mm. FLAIR sequence was performed with: repetition time msec/echo time msec, 8500-10000/85-135; section thickness, 2.5–6.0 mm; intra-section spacing, 0.36–1.02 mm.

### Image Preprocessing and Automatic Segmentation

Based on T1, T1C, T2 and FLAIR images, we aimed to automatically segment the images into five classes: the non-tumor region and four tumor subregions including necrosis, edema, non-enhancing area, and enhancing area. Detailed definition of these subregions can be found in^15^. First, the images were preprocessed, encompassing N4 correction of bias field^16^, skull stripping, image resampling, rigid registration using T1C image as a template with the mutual information similarity metric, and intensity normalization by histogram matching. All preprocessing was done using the ITK software tool^17^.

Then, a voxel-wise random forest model with a conditional random field spatial regulation was used to classify the images into five classes. The random forest can efficiently solve multi-class correlated-feature problems with a probabilistic output instead of hard classification labels. To train the random forest classifier, real patient MR data sets from the 2015 brain tumor segmentation challenge (BRATS 2015) were used. For each training sample 100,000 randomly sampled voxels were used to train the random forest, while for each testing sample all voxels were used. The number of trees was set to 100. The automatic segmentation was implemented using Matlab software. Details of the automatic segmentation method can be found in Supplementary Method 1.

### High-throughput Radiomics Features Extraction

Within the segmented tumor subregions high-throughput imaging features can be extracted. To fully characterize the intra-tumor heterogeneity, we extracted 45792 first-order and high-order texture features from 6 extraction subregions, including necrosis, enhancing area, non-enhancing area, edema, solid core (the whole tumor except edema) and whole tumor. The features extracted were summarized in Supplementary Table 1.

To test the effects of image standardization parameters, the features were extracted at different combinations of voxel size, quantization method and gray level, as shown in Supplementary Figure 1. Before features extraction all voxels were isotropically rescaled into three vixel sizes of 1, 2, and 3 mm. From all 6 subregions in 4 modalities, totally 864 first-order features were extracted for each patient, where each first-order feature had 3 measures at voxel size = 1, 2, and 3 mm. Before the high-order texture feature extraction, the intensities were required to be quantized to a certain number of gray levels. Here we tested three quantization methods (uniform quantization, the equal-probability quantization, and the Lloyd-Max quantization^18^) and four gray levels including (32, 64, 128 and 256). Therefore, we had 36 combinations of different parameter settings, yielding 36 measures for each high-order texture feature. Totally we extracted 44928 high-order texture features from all 6 subregions in 4 modalities.

### Statistical Analysis

All statistical analysis was done with R software, version 3.4.0 (https://www.r-project.org/) and X-tile software, version 3.6.1 (Yale University School of Medicine, New Haven, Conn)^19^. The statistical significance levels were set at 0.05.

#### Clinical Characteristics and survival

The differences in age, sex, tumor volume, KPS, mean follow-up time and mean survival between the training and the validation data sets were assessed using an independent sample *t* test, Mann-Whitney *U* test or ${\chi }^{2}$ test, where appropriate.

#### Reproducibility of Radiomics Features

The reproducibility was assessed by measuring the agreement in features extracted at different parameter settings using the overall concordance correlation coefficient (OCCC)^20^. OCCC was designed to assess the agreement among multiple observations, where it was the case here of multiple parameter settings.

For each first-order feature, an OCCC index can be computed among its 3 measures at voxel size = 1, 2, and 3 mm. Each high-order texture feature had 36 measures corresponding to 36 parameters settings, varying 3 voxel sizes, 3 quantization methods and 4 gray levels. Instead of computing the OCCC over all possible combination of the 36 measures, we separately assessed the effect of varying one parameter on a feature while fixing the other two parameters. The advantage was that we can separately quantify the effect of each parameter on the reproducibility of each high-order feature. The R packages epiR was used for calculating OCCC.

#### Prognostic Performance of Radiomics Features

The univariate prognostic performance of each feature measure was assessed using the concordance index (C-Index), a generalization of the area under the receiver operating characteristic (ROC) curve (AUC)^21^. Univariate C-Index was calculated for each measure of a feature. Each first-order texture feature had 3 measures, so 3 C-Indices were calculated. Each high-order texture feature had 36 measures and therefore 36 C-Indices can be calculated. The R packages Hmisc was used for calculating C-Index.

#### Feature Selection and Multiparametric Radiomics Signature Construction

From all 864 first-order features, features with OCCC $\ge$ 0.85 were considered as reproducible and were selected. While from all high-order features, we aimed to select features with high OCCCs in the tests. For each high-order feature if 10 (or more) out of 33 OCCCs were greater than 0.85, that feature was considered as reproducible against the vary parameters and were selected. We next selected features with better prognostic value. Features with univariate C-Index $\ge \,0.60$ (positive association) or $\le 0.40$ (negative association) were selected as better prognostic factors. Furthermore, correlation coefficient between each pair of features was calculated. For feature pair with correlated coefficient $\ge 0.90$, the more prognostic feature was retained and the other was removed. Finally, the selected features were regarded as reproducible, prognostic and nonredundant.

Based on the selected radiomics features, we aimed to construct a multiparametric signature using multivariate Cox regression for OS prediction. Here multiparametric meant that the features used for signature construction were extracted at multiple image standardization parameters. Note that there were more features than patients. According to the Harrell guideline^22^, a multivariate regression model is likely to be reliable when the number of included covariates is less than 1/10 of samples. Therefore, the least absolute shrinkage and selection operator (LASSO) Cox regression model was used on the training data set for prognostic signature construction^23^. Depending on the weight $\lambda$, LASSO shrinks all regression coefficients towards zero and set the coefficients of many irrelevant features exactly to zero. To find an optimal $\lambda$, 10-fold cross validation with minimum criteria was employed, where the final value of $\lambda$ gave minimum cross validation error. The retained features with nonzero coefficients were used for regression model fitting and combined into a radiomics signature. The R package glmnet was used for LASSO Cox regression modeling.

#### Fixed-parameter Radiomics Signatures Construction

The fixed-parameter radiomics signatures were constructed similarly. At each fixed parameter settings (voxel size, quantization method and gray level), we had 1536 features (288 first-order features and 1248 high-order features). Features with C-Index $\ge \,0.60$ or $\le 0.40$ were selected. Then, the LASSO Cox model was used on the training data set for signature construction. As we totally had 36 parameter settings, finally 36 fixed-parameter radiomics signatures were constructed.

#### Validation of the Multiparametric Radiomics Signature

The association of the multiparametric radiomics signature with OS was assessed on the training data set and validated on the validation data set by using Kaplan-Mier survival analysis. The C-Index was used to assess the prognostic performance of the radiomics signature. According to a threshold estimated based on the radiomics score by using an optimal cutpoint analysis with X-tile software^19^, patients were stratified into high-risk and low-risk groups. The threshold was calculated on the training data and tested on the validation data. A weighted log-rank test (G-rho rank test, rho = 1) was used to test the significant difference between the high- and low-risk groups. The R package survcomp was used for the survival analysis.

#### Prognostic Performance Comparison of Multiparametric and Fixed-parameter Signatures

The prognostic performance of the multiparametric signature was compared with 36 fixed-parameter signatures and 3 conventional prognostic factors such as age, Karnofsky Performance Score (KPS) and tumor volume. The association of each fixed-parameter signature and each conventional factor with OS were assessed on the training data set, and further tested on the validation data by using Kaplan-Mier survival analysis. The prognostic performance comparisons were conducted on the validation data set.

## Results

There was no significant difference in clinical and follow-up data between the training and validation data sets (P = 0.558 to 0.977).

### Segmentation Results

**Figure 1:**
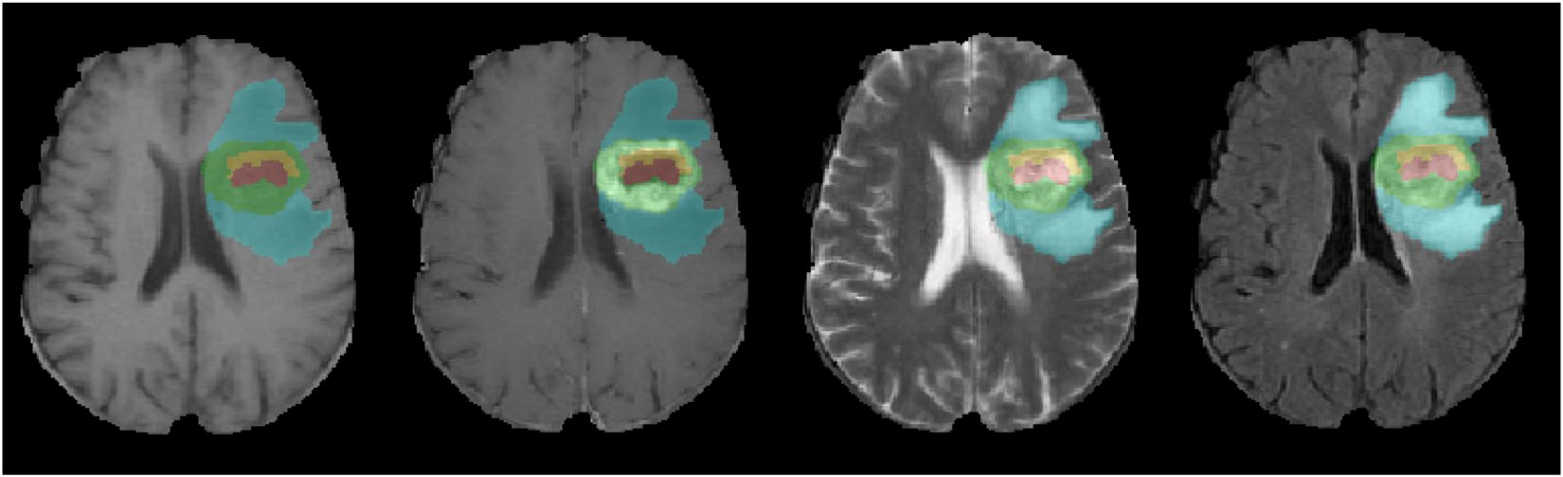
Segmentation results overlap on T1, T1C, T2 and FLAIR images.

The segmentation performance was reported by the BRATS online evaluation system^15^ and summarized in Supplementary Table 2. One example of the segmentation results from our SYSUCC validation data set is shown in Fig. 1. Subregions were shown in red (necrosis), green (enhancing region), yellow (non-enhancing region) and blue (edema). All images were skull-stripped and registered using T1C as a template.

### Effects of Image Standardization Parameters on Feature Reproducibility

**Figure 2:**
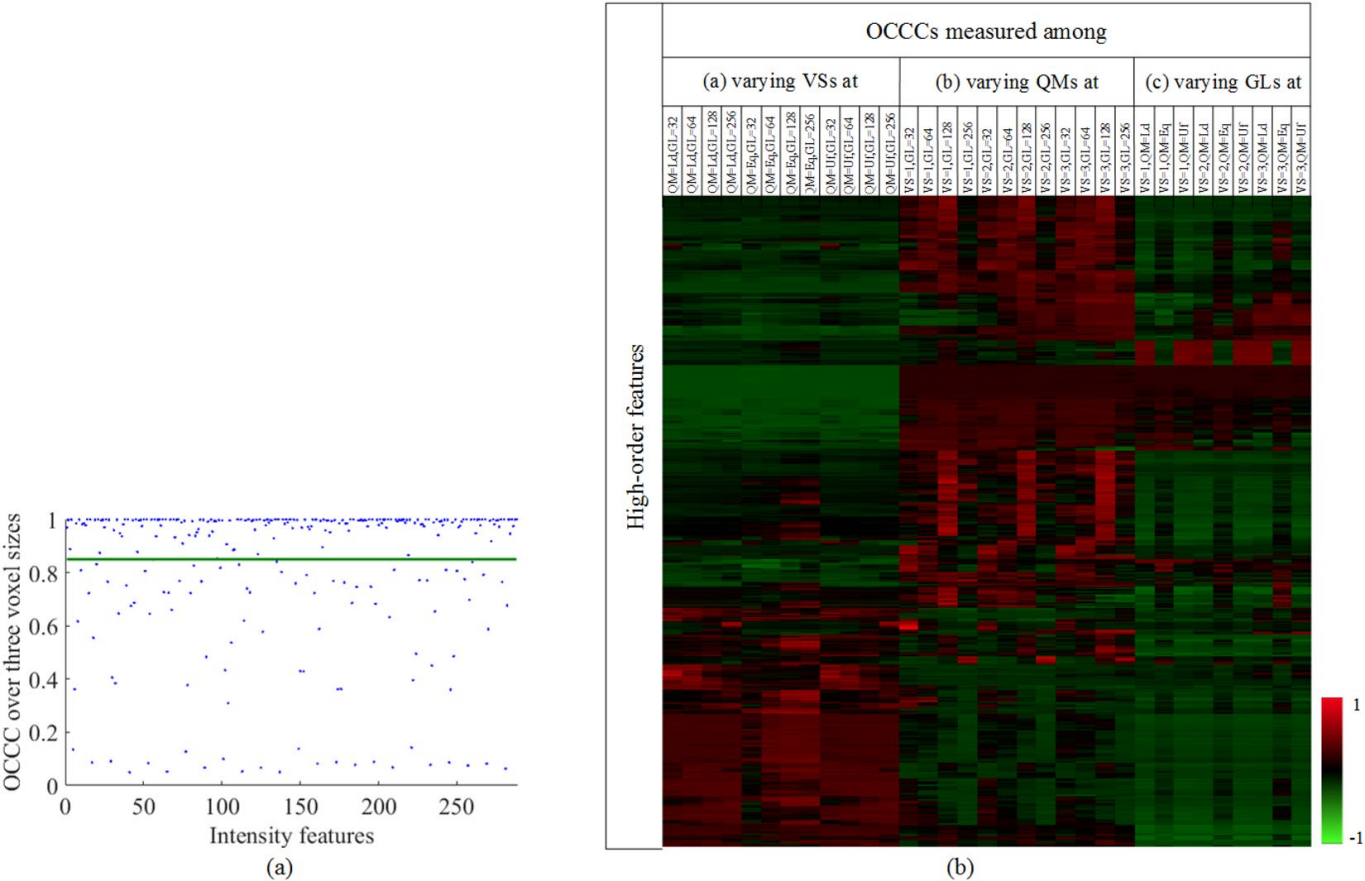
(**a**) OCCCs of the first-order features. (**b**) OCCC heat map of high-order features, where OCCCs (z-score: −1 to 1) were clustered along y axis. The brighter the red (green) color, the higher (lower) the OCCC value. The OCCCs were calculated over different image standardization parameters. Part (**a**) OCCCs measured among three voxels sizes with fixed quantization method and gray level. Part (**b**) OCCCs measured among three quantization methods with fixed voxel size and gray level. Part (c) OCCCs measured among four gray levels with fixed voxel size and quantization method. VS, QM, GL, Uf, Eq and Ld are short for voxel size, quantization method, gray level, uniform, equal-probability, and Lloyd-Max, respectively.

Figure 2a shows the OCCCs of the first-order texture features, indicating the effect of voxel size on the reproducibility of the first-order features. The green line indicates OCCC = 0.85. The OCCC indices of 195 first-order features were greater than 0.85, demonstrating the high reproducibility of these features against varying voxel sizes.

Figure 2b shows the OCCC map for the high-order texture features. The effects of voxel size, quantization method and gray level on OCCCs (i.e. feature reproducibilities) were separately measured in part (a,b,c) in Fig. 2b. We can see no feature had high OCCC in all 33 tests (there is not a “red row” across all three parts (a,b,c)). It indicated that no feature was highly reproducible against the three tested image standardization parameters. It can be observed that the high OCCC values were naturally clustered (red areas) along the horizontal axis. Specifically, there are many red rows across about 10 or more tests in parts (a,b,c). Therefore, the selected high-order feature with 10 or more OCCCs greater than 0.85 was more likely to be reproducible against one varying parameter with the other two parameters fixed.

### Effects of Image Standardization Parameters on Feature Prognosis

**Figure 3:**
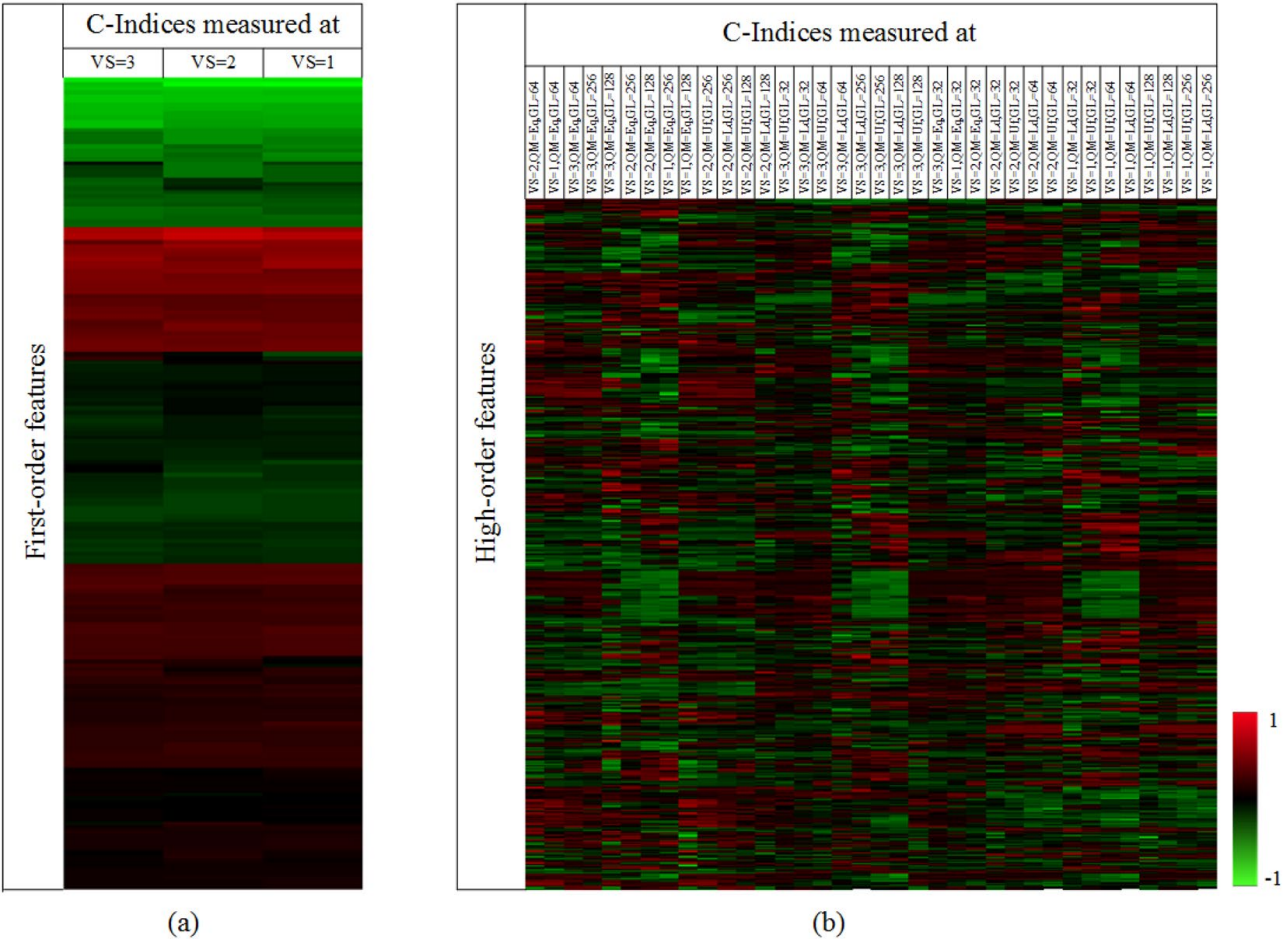
(**a**) C-Index heat map of reproducible first-order texture features. (**b**) C-Index heat map of reproducible high-order texture features. C-Indices (z-score: −1 to 1) were clustered along both x and y axes. The brighter the red (green) color, the higher (lower) the C-Index.

Figure 3 are heat maps showing the C-Indices for the selected first- and high-order features, respectively. The three columns in Fig. 3a indicate the C-Indices for all selected first-order features measured at voxel size = 1, 2, and 3 mm, respectively. Each column in Fig. 3b indicates C-Indices for all selected high-order features measured at one of 36 parameter settings. It was clear that the image standardization parameters had impact on the C-Indices.

### Construction of the Multiparametric Radiomics Signature

884 features (195 first-order features and 689 high-order features) were selected as reproducible features, as summarized in Supplementary Table 3. From these features, the most prognostic 163 features were selected, including 12 first-order features and 151 high-order features. After correlation removal, 97 out 163 features remained for the multivariate model building (Supplementary Figure 2 shows the heat map for the spearman correlation coefficients of the 163 features). The optimal $\lambda$ selection in LASSO Cox regression model for the multiparametric signature is shown in Supplementary Figure 3, where the Cox partial likelihood deviance was plotted versus $\mathrm{log}(\lambda )$. At the optimal $\lambda =0.179$, as shown at the dotted vertical line, the 10-fold cross validation error was minimum. The selected $\lambda$ resulted in 4 features with nonzero regression coefficients. By linearly combining the four features weighted by their coefficients, the multiparametric radiomics signature can be constructed. The multiparametric radiomics score can be computed as

1 $$\begin{array}{l}\text{Rad}\,\text{Score}=-0.6708511\cdot {f}_{1}+0.6671869\cdot {f}_{2}+0.5214041\cdot {f}_{3}-0.1500439\cdot {f}_{4},\end{array}$$

where ${f}_{1},{f}_{2},{f}_{3},{f}_{4}$ were the four selected features, as shown in Table 2 with their weights and image standardization parameters. Here GLCM_IDMN was short for Inverse Difference Moment Normalized calculated based on Gray Level Co-occurrence Matrix. GLRLM_HGRE meant High Gray-level Run Emphasis calculated based on Gray Level Run Length Matrix. GLSZM_GLN represented Gray Level Non-uniformity based on Gray Level Size Zone Matrix. GLCM_IMC2 represented Informational Measure of Correlation based on Gray Level Co-occurrence Matrix. The univariate C-Indices were calculated based on validation data. Based on the radiomics score of patients in the training data set, the optimal cutoff calculated by the X-tile plot was −19.57. Then, patients in both the training and validation data sets were stratified into low-risk ($Rad\_Score < -19.57$) and high-risk ($Rad\_Score\ge -19.57$) groups.

**Table 2:** The image features selected by LASSO Cox model for construction of multiparametric radiomics signature. The features were ranked by their contribution to the signature. The feature was named as *modality*_*region*_*matrix*_*title*, where *title* can be found in Supplementary Table 1. Note that there were two different calculations for GLCM_IMC, which can be found in^4^. Here GLCM_IMC2 indicated the second calculation listed in^4^.

No.	Feature	Weight	Extraction Parameters	C-Index on Validation Data
${f}_{1}$	T1_wholetumor_GLCM_IDMN	−0.67085113	VS = 1, QM = Ld, GL = 128	0.397
${f}_{2}$	T1C_solidecore_GLRLM_HGRE	0.66718691	VS = 1, QM = Ld, GL = 32	0.582
${f}_{3}$	T1_wholetumor_GLSZM_GLN	0.52140412	VS = 1, QM = Eq, GL = 32	0.605
${f}_{4}$	T1C_nonenhancing_GLCM_IMC2	-0.15004393	VS = 1, QM = Eq, GL = 64	0.401

### Construction of the Fixed-parameter Radiomics Signatures

The selected features, their individual nonzero coefficients, image standardization parameters and corresponding optimal $\lambda$ in the LASSO models of the 36 fixed-parameter radiomics signature are listed in Supplementary Table 4. The formulas for calculating the radiomics score can then be known by linearly combining the features weighed by their coefficients. The optimal cutoff points calculated by using the X-tile plot are also listed in Supplementary Table 4. The number of nonzero features varied from 1 to 7 among the 36 signatures. The type of nonzero features also varied greatly across these signatures.

### Validation of the Multiparametric Radiomics Signature

**Figure 4:**
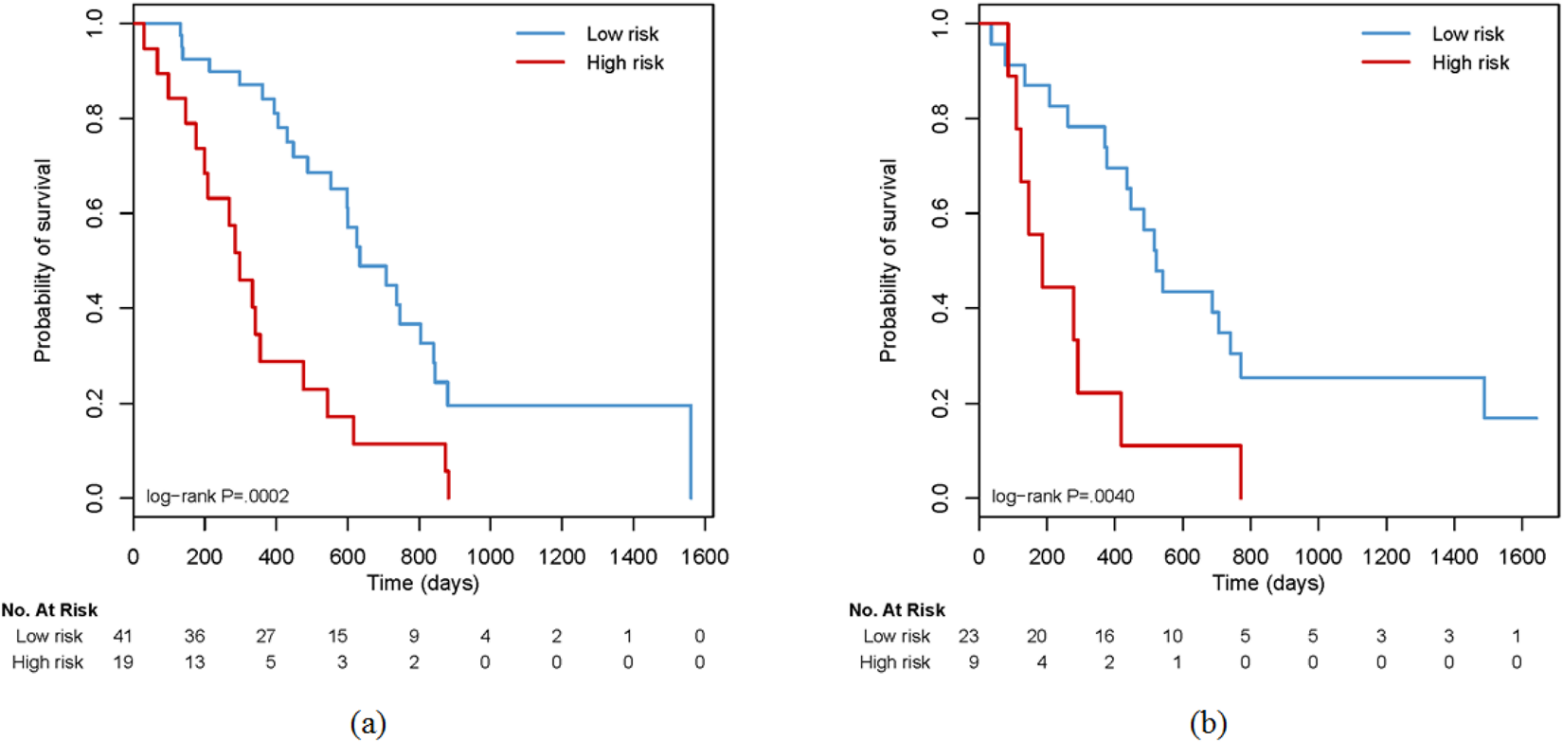
Kaplan-Meier survival curves for patients in the training data set (**a**) and the independent validation data set (**b**). The patient cohorts were stratified into low- and high-risk groups according to the radiomics score. The significant association of the radiomics signature with overall survival was confirmed in both data sets. The numbers of patients at risk for each time step are shown in the bottom.

The C-Index of the multiparametric signature achieved 0.726 (95% confidence intervals [CI]: 0.698, 0.754) for the training data, and 0.705 (95% CI: 0.672, 0.738) for the independent validation data. It demonstrated the prognostic performance of the model. The association of the multiparametric signature with OS was significant in the training data set (P $<$ 0.001, hazard ratio [HR] = 3.063, 95% CI: 1.643, 5.721). The significant association was confirmed in the validation data set (P = 0.004, HR = 3.292, 95% CI: 1.401, 7.702), as shown in Fig. 4. The log-rank test revealed the significant difference in OS distributions of the low- and high-risk groups.

### Prognostic Performance Comparison

The prognostic performance for the 36 fixed-parameter signatures are listed in Supplementary Table 5. The Kaplan-Meier curves for OS in the validation data set using the 36 fixed-parameter radiomics signatures were shown in Supplementary Figure 4. The patients were stratified by the cutoff point of each fixed-parameter signature. 12 fixed-parameter signatures succeeded to stratify the patients in the validation data set into high-risk and low-risk groups, while the others failed. The prognostic value of the multiparametric radiomics signature and conventional factors in the validation data is shown in Table 3. The multiparametric signature outperformed all the fixed-parameter signatures and all the conventional factors.

**Table 3:** Prognostic value comparison of the proposed multiparametric radiomics signature and conventional factors on the validation data. The data in parentheses are 95% confidence intervals.

Factors	C-Index	*P* Value	Hazard Ratio
Multiparametric Radiomics Signature	0.705	0.004	3.292 (1.401, 7.702)
Age	0.595	0.350	1.664 (0.814, 4.261)
KPS	0.605	0.220	2.090 (1.082, 8.232)
Tumor Volume	0.603	0.235	1.851 (1.013, 9.633)

## Discussion

This study presented a fully-automatic multiparametric radiomics model for preoperative prediction of OS in GBM patients. This study had several unique features and interesting findings as follows.

First, this study offered a fully-automatic way to build a multiparametric imaging signature for OS prediction, where the most reproducible and prognostic features and their corresponding image standardization parameters can be determined automatically. In fully-automatically models, the effect of image standardization parameters such as voxel size, quantization method and gray level on reproducibility and prognostic performance are still unclear. Most radiomics models extracted feature at fixed image standardization parameters^5–9^, which may not be optimal. Different from most existing radiomics models, the presented model allowed for extraction of high-throughput features with different parameter settings, and further selected the most reproducible and prognostic features for signature construction. This may offer potential to improve the model reproducibility and prognostic power.

Second, it was demonstrated that voxel size, intensity quantization method and gray level had significant influence on the reproducibility of imaging features (Fig. 2). It was also found that no texture feature was stable over all tested parameter settings. The reproducibility relied on parameter settings. This was partially similar with the effect of the reconstruction parameters on the PET imaging feature reproducibility^14^. However, they only investigated a relatively small number of features. In our results, part of GLRLM-based texture features were more reproducible against varying voxel sizes, but less reproducible against different quantization methods and gray levels. In contrast, most GLRLM LGRE, GLRLM LRLGE and GLRLM SRLGE-based texture features (Find the abbreviations of the image features in Supplementary Table 1) were more robust against varying quantization methods and gray levels, but less robust over varying voxel sizes. Most GLCM-based texture features were stable over different quantization methods, but had low repeatability at varying voxel sizes and gray levels. We also observed that a number of NGTDM-based texture features achieved high reproducibility at gray level of 128 (three short red vertical lines in Fig. 3), but had relatively low reproducibility at other parameter settings. It was clear that one feature may be highly reproducible under certain parameter settings, but acted differently under other parameter settings. This highlighted the need to extract features at their own optimal parameter settings rather than at fixed parameters.

Third, the image standardization parameters also had significant influence on the prognostic performance of the extracted features. Different from the CT reconstruction parameters^12^, we observed that the influence of image standardization parameters on feature prognosis did not exhibit regular patterns. We also observed that no feature was highly prognostic in all parameter settings, and no parameter setting was optimal for all features. Therefore, our findings suggest that in radiomics study it is better to extract image features at multiple image standardization parameter settings according to their reproducibility and prognostic performance.

Fourth, a multiparametric radiomics signature was constructed and succeeded to stratify GBM patients into high- and low-risk groups with significant differences in OS. It outperformed all 36 fixed-parameter signatures in terms of prognostic performance. We believe the reason was that the features used for building the multiparametric signature were more informative than the traditional fixed-parameter features. On the other hand, 12 out of 36 fixed-parameter signatures also succeeded to stratify the patients into high- and low-risk groups. The best fixed-parameter signature was the 11th one built at voxel size = 1mm and gray level = 128 with uniform quantization. Its C-Index on validation data set was 0.701 (P = 0.008, HR = 4.297, 95% CI: 1.879, 9.830), which was slightly lower than the multiparametric signature. Although the performance of the best fixed-parameter signature may approximate the multiparametric signature, it could be a heavy and tedious task to find out the best fixed-parameter signature among a number of candidates. The proposed method to obtain the multiparametric signature was more straightforward.

Fifth, the identified multiparametric signature comprised 4 image features shown in Table 2. Interestingly, they were all high-order texture features, extracted with different image standardization parameters combinations from the whole tumor in T1 images, and from the tumor core and non-enhancing area in T1C images. We believe it was because high-order texture features reflected more nonrandom patterns (more reproducible) and captured more imaging heterogeneity (more prognostic) compared with first-order features. Specifically, ${f}_{1}$ measured the textural smoothness of the whole tumor area; ${f}_{2}$ characterized the distribution of gray levels of runs in the solid tumor core; ${f}_{3}$ described the texture homogeneity of the whole tumor area; ${f}_{4}$ quantified the informational correlation of voxel pairs in the non-enhancing area. This result was similar to several previous studies in^5,9^. However, the selected 4 features were different from the results in previous work^8,9^. One reason was that previous studies did not use multiple image standardization parameters. Note that the feature ${f}_{4}$ from non-enhancing area was finally selected, indicating its better univariate prognostic value. Similarly, the study in^24^ demonstrated that morphologic imaging features and hemodynamic parameters obtained from the non-enhancing area correlated with patient survival. Our finding implied that the non-enhancing area could provide useful prognostic information. One possible reason was that the texture of non-enhancing area may reflect the aggressiveness and invasiveness of the tumor. From the radiomics hypothesis, imaging heterogeneity could be the expression of genetic heterogeneity which could indicate poorer prognosis^10^. The interpretation of association between radiomics features and genetic characteristics is still challenging, which is related to complex underlying biological processes^25^. To address this radiogenomics studies are required in future.

Sixth, in addition to assess the univariate reproducibility and prognostic power of individual feature, the impact of different voxel sizes, quantization methods and gray levels on the prognostic performance of radiomics signatures was also investigated in detail, which has never been studied. The results in Supplementary Table 5 and Supplementary Figure 4 indicated that fixed-parameter signatures based on voxel size of 1mm were found to perform better than those built at larger voxels. For the multiparametric signature, all 4 non-zero features were also built at voxel size of 1mm. This finding was similar with previous radiomics studies based on CT images^12^. One possible reason was that larger voxel size could affect the spatial distributions of the intensities, thusly reduce the value of high-order texture features extracted.

This study still had several limitations. First, this was a retrospective study with relatively small patient cohort, although independent validation cohort was used. The bias was controlled and patients with loss of follow-up were excluded. In future, large-scale multicenter study is required. Second, the association between imaging features and underlying genetic characteristics was not assessed. In future, our radiogenomics study will involve several well-studied genomic signatures for GBM, such as the O^6^-methylguanine-DNA methyltransferase (MGMT) methylation status and the isocitrate dehydrogenase (IDH) 1/2 mutations.

In conclusion, this paper presented a radiomics model that offered a fully-automatic workflow to generate reproducible and prognostic multiparametric imaging signature. The proposed multiparametric signature predicted OS in GBM patients with better performance compared with the fixed-parameter radiomics signatures and conventional prognostic factors. Despite the limitations, the proposed method had the potential to facilitate the preoperative patient care and made a step forward radiomics-based precision medicine of GBM patients.

## Electronic supplementary material

Supplementary Information
