# Supplementary material for: A Fully-Automatic Multiparametric Radiomics Model: Towards Reproducible and Prognostic Imaging Signature for Prediction of Overall Survival in Glioblastoma Multiforme
Source: Sci Rep. 2017 Oct 30;7:14331. doi: 10.1038/s41598-017-14753-7 (PMC5662697; doi:10.1038/s41598-017-14753-7)
Supplement: Supplementary file 1 — Supplementary Information [file 41598_2017_14753_MOESM1_ESM.pdf]

Supplementary Material
A Fully-Automatic Multiparametric Radiomics Model: Towards Reproducible and Prognostic Imaging Signature for Prediction of Overall Survival in Glioblastoma Multiforme

Qihua Li[1,+], Hongmin Bai[2,+], Yinsheng Chen[3,+], Qiuchang Sun[1], Lei Liu[1], Sijie Zhou[2], Guoliang Wang[2], Chaofeng Liang[4,*], and Zhi-Cheng Li[1,+,*]

[1]Institute of Biomedical and Health Engineering, Shenzhen Institutes of Advanced Technology, Chinese Academy of Sciences, Shenzhen, China.
[2]Department of Neurosurgery, Guangzhou General Hospital of Guangzhou Military Command, Guangzhou, China.
[3]Department of Neurosurgery/Neuro-oncology, Sun Yat-sen University Cancer Center, State Key Laboratory of Oncology in South China, Collaborative Innovation Center for Cancer Medicine, Guangzhou, China.
[4]Department of Neurosurgery, The 3rd Affiliated Hospital of Sun Yat-sen University, Guangzhou, China
[*]Corresponding author: Chaofeng Liang (lcfjeff@163.com) and Zhi-Cheng Li (zc.li@siat.ac.cn).
[+]Qihua Li, Hongmin Bai, Yinsheng Chen and Zhi-Cheng Li contributed equally to this work.

Supplementary Method 1

Automatic Segmentation of Multiregional GBM

Based on T1, T1C, T2 and FLAIR images, we aimed to segment the image into five classes: the non-tumor region (label 0) and four tumor subregions including necrosis (label 1), edema (label 2), non-enhancing area (label 3), and enhancing area (label 4). We used a voxel-wise random forest method to classify the images into five classes. Random forest is able to provide the feature importance measures directly [1]. The first step was feature extraction (for segmentation, not for radiomics analysis). For voxel $i \in P$ where P denote the voxel set, the features extracted from each MR modalities comprised 1 intensities, 6 first-order textures including mean, variance, skewness, kurtosis, energy and entropy, 16 Gabor texture features, 2 symmetric features, and 24 context features.

For each voxel i in four MR modalities we obtained a 196-dimensional feature vector $\mathbf{x}_i$ as

$$\mathbf{x}_i = [\mathbf{x}_{i,T1}, \mathbf{x}_{i,T1C}, \mathbf{x}_{i,T2}, \mathbf{x}_{i,FL}], \tag{1}$$

where $\mathbf{x}_{i,T1}, \mathbf{x}_{i,T1C}, \mathbf{x}_{i,T2}$, and $\mathbf{x}_{i,FL}$ denoted the feature vectors extracted from the four MR modalities, respectively. Then, we used random forest as the classifier that output for every voxel i a probability $Pr(y_i|\mathbf{x}_i)$ corresponding to every tissue type $y_i \in \{0, 1, 2, 3, 4\}$.

During the classification, the importance of each feature $x_i \in \mathbf{x}_i$ can be used to assess their contributions to the classification task [2] and be computed as

$$w_{mod} = \frac{1}{W} \sum_{x \in \mathbf{x}_{mod}} w(x), \tag{2}$$

where mod denoted a specific MR modality, $w(x)$ was the importance for feature x that belongs to $\mathbf{x}_{mod}$, and W was the sum of the importance for all features. Here we had $w_{T1} + w_{T1C} + w_{T2} + w_{FL} = 1$.

Then, the final segmentation can be formulated by minimizing a second-order CRF cost function as

$$E_i = \sum_{i \in P} D(y_i, \mathbf{x}_i) + \sum_{i \in P, j \in N_i} V(y_i, y_j, \mathbf{x}_i, \mathbf{x}_j), \tag{3}$$

where the singleton potential D and the pairwise potential V denoted respectively the data cost and the prior smoothness cost. $(\mathbf{x}_i, y_i)$ was the feature-label pair for voxel i, and N_i was the neighbors of voxel i. Here we used 26-neighborhood system. The data cost D only depended on $(\mathbf{x}_i, y_i)$, representing the penalty of assigning label y_i to voxel i. D was defined as the local negative log-likelihood as

$$D(y_i, \mathbf{x}_i) = -\ln Pr(y_i|\mathbf{x}_i). \tag{4}$$

The pairwise potential V posed spatial smoothness constrain in neighboring voxels. Taking into account the different importance of features from different modalities, V was given by a weighted sum of four smoothness functions corresponding to the four modalities as

$$V = w_{T1}V_{T1} + w_{T1C}V_{T1C} + w_{T2}V_{T2} + w_{FL}V_{FL}. \tag{5}$$

Inspired by the work given by Boykov et al. in [3], V_{T1} was defined as

$$V_{T1} = \lambda_{i,j} \cdot \exp\left(-\frac{(I_i - I_j)^2}{2\sigma^2}\right) \cdot \frac{\delta(y_i, y_j)}{\|i - j\|}, \tag{6}$$

where

$$\delta = \begin{cases} \text{A} & \text{if } y_i = 0, y_j = 1, 4 \text{ or } y_j = 0, y_i = 1, 4 \\ 1 & \text{otherwise if } y_i \neq y_j \\ 0 & \text{otherwise} \end{cases} . \tag{7}$$

In Eq.(6), $\lambda_{i,j}$ was a weighting factor. I was the intensity. σ was the intensity variance within the local neighborhood system. The function penalized a lot for feature discontinuous between neighbors with similar intensities when $|I_i - I_j| < \sigma$. If the intensity difference was large, i.e. $|I_i - I_j| > \sigma$, the penalty becomes small. The δ function penalized different labels between neighbors, especially for the case of necrosis or enhancing area neighboring healthy tissue ($A = 3.5$ here). Then, V_{T1C}, V_{T2}, V_{FL} can be defined in similar formulations. Finally, the CRF cost function in Eq.(3) was optimized using the graph-cuts method [4].

Supplementary Table 1: A Summary of The high-throughput radiomics features extracted. Here the high-order texture features were extracted using several different methods, including the gray-level co-occurrence matrix (GLCM), gray-level run length matrix (GLRLM), gray level size zone matrix (GLSZM) and neighborhood gray-tone difference matrix (NGTDM) methods. The calculation details of these features can be found in [5]. In total, 45792 features were extracted, consisting of 864 first-order features, 18144 GLCM features, 11232 GLRLM features, 11232 GLSZM features, and 4320 NGTDM features. The number of basic types of first-order, GLCM, GLRLM, GLSZM and NGTDM features were 12, 21, 13, 13, and 5, respectively. Note that there were two different calculations for both GLCM_Homogeneity and GLCM_Informational Measure of Correlation, which can be found in [5]. The signature feature f_4 used the second calculation of GLCM_Informational Measure of Correlation in [5]. The number of features extracted in each class can then be calculated. For example, GLRLM features were extracted with 36 parameter combinations from 6 regions and 4 MR modalities, so in total we had $13 \times 36 \times 6 \times 4 = 11232$ GLRLM features.

**Feature Classes**		**Feature Names**
First-order Texture Features		MaxValue, MedianValue, MinValue, MeanValue, Energy, Entropy, Variance, Kurtosis, Root Mean Square, Skewness, Standard Deviation, Mean Absolute Deviation
High-order Texture Features	GLCM Features	Contrast, Correlation, Difference Entropy, Entropy, Informational Measure of Correlation, Sum Average, Sum Entropy, Sum Variance, Variance, Difference Variance, Autocorrelation, Cluster Prominence, Energy, Cluster Shade, Dissimilarity, Inverse Difference Normalized, Homogeneity, Maximum Probability, Inverse Difference Moment Normalized
	GLRLM Features	Short Run Emphasis, Long Run Emphasis, Gray-Level Non-uniformity, Run-Length Non-uniformity, Low Gray-Level Run Emphasis, High Gray-Level Run Emphasis, Short Run Low Gray-Level Emphasis, Short Run High Gray-Level Emphasis, Gray-Level Variance, Long Run Low Gray-Level Emphasis, Run-Length Variance, Long Run High Gray-Level Emphasis, Run Percentage
	GLSZM Features	Small Zone Emphasis, Large Zone Emphasis, Gray-Level Non-uniformity, Zone-Size Non-uniformity, Low Gray-Level Zone Emphasis, High Gray-Level Zone Emphasis, Small Zone Low Gray-Level Emphasis, Small Zone High Gray-Level Emphasis, Gray-level Variance, Large Zone Low Gray-Level Emphasis, Zone-Size Variance, Large Zone High Gray-Level Emphasis, Zone Percentage
	NGTDM Features	Coarseness, Contrast, Busyness, Complexity, Strength

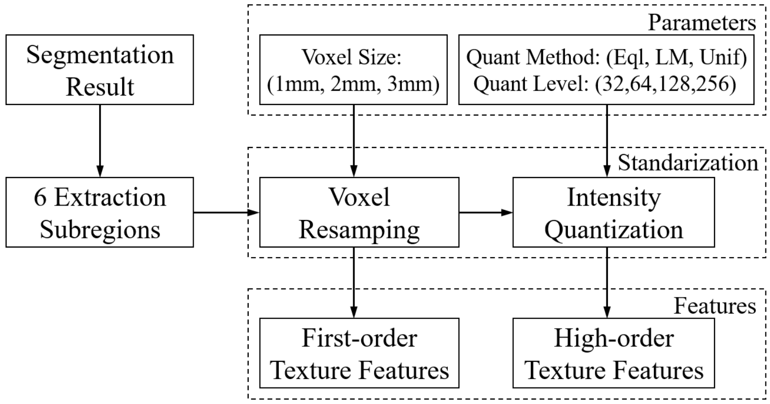

Supplementary Figure 1: Extraction of high-throughput multiparametric radiomics features. To test the effects of image standardization parameters, the features were extracted at different combinations of voxel sizes (1, 2, and 3mm), quantization methods (uniform quantization, the equal-probability quantization, and the Lloyd-Max quantization) and gray levels (32, 64, 128 and 256).

Supplementary Table 2: Segmentation results on subset of BRATS2015 test data. For BRATS, three tumor regions were evaluated, including the whole tumor (tumor core and edema), the tumor core (necrosis, enhancing area and non-enhancing area) and the active tumor (enhancing area). For each tumor region, three metrics were calculated via the BRATS online evaluation system, including the Dice score, the sensitivity and the specificity. Each measure was given as median and range.

Tumor Region	DICE score	sensitivity	specificity
whole tumor	0.824, 0.370	0.861, 0.329	0.929, 0.565
tumor core	0.752, 0.281	0.809, 0.357	0.824, 0.352
active tumor	0.709, 0.445	0.811, 0.292	0.845, 0.473

Supplementary Table 3: A Summary of the Reproducible Features Selection. VS, QM and GL are short for voxel size, quantization method and gray level, respectively.

Feature Classes		Reproducible First-order Features Against	Reproducible high-order Features Against			
		VS	VS	QM	GL	Summary
MRI Modalities	T1	47	48	94	70	156
	T1C	49	53	117	63	182
	T2	50	50	126	68	193
	FLAIR	49	50	91	69	158
Tumor Subregions	Necrosis	30	1	111	75	120
	Enhancing	34	48	65	30	119
	Nonenhancing	28	0	79	69	106
	Edema	32	51	60	32	117
	Core	36	51	64	33	120
	Whole Tumor	35	50	49	31	107
Texture Types	GLCM	-	120	128	140	291
	GLRLM	-	49	112	52	172
	GLSZM	-	32	131	54	169
	NGTDM	-	0	57	24	57
Summary	-	195	689			

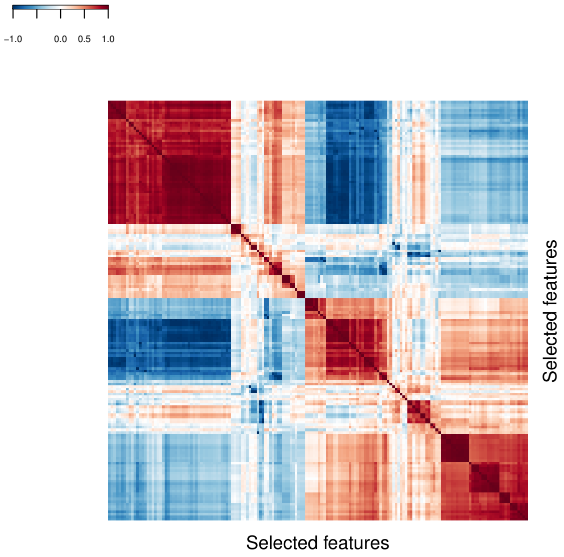

Supplementary Figure 2: Heat map of the correlation coefficients of the selected 164 features. The coefficients (z-score: -1 to 1) were clustered. The brighter the red (blue) color, the higher (lower) the correlation.

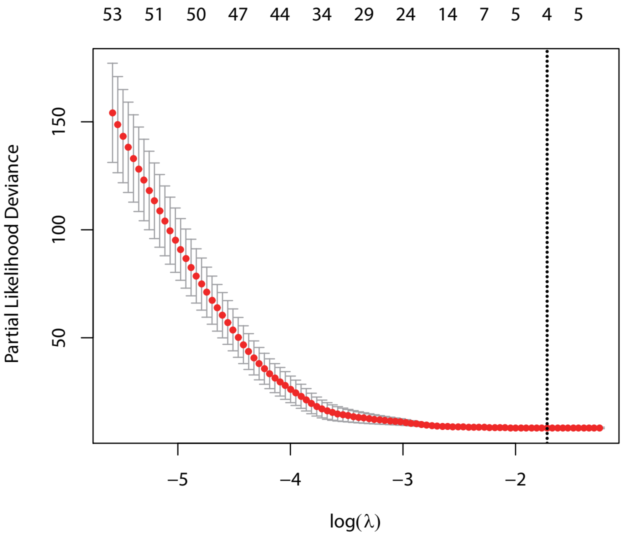

Supplementary Figure 3: The optimal λ selection in LASSO Cox regression model for the multiparametric radiomics signature. The partial likelihood deviance was plotted versus $\log(\lambda)$. The corresponding numbers of nonzero regression coefficients were shown at the top. The dotted vertical line was plotted at the optimal λ of 0.179 ($\log(\lambda) = -1.720$), generating 4 nonzero coefficients.

Supplementary Table 4: The results for construction of all 36 fixed-parameter radiomics signatures. The image standardization parameters, the selected features and corresponding coefficients, the optimal λ ($\log(\lambda)$ shown) and the optimal cutoff values are listed. VS, QM, GL, Uf, Eq and Ld are short for voxel size, quantization method, gray level, uniform, equal-probability, and Lloyd-Max, respectively.

Index	Parameters			Selected Features	Coefficients	log(λ)	Cutoff
	VS	QM	GL				
1	1	Ld	32	T1c_wholetumor_Energy	0.13966959	-1.638711	0.1857687
				T1_wholetumor_GLCM_IMC1	0.12650752		
				T1_wholetumor_GLSZM_GLN	0.03110558		
				T1c_solidcore_GLRLM_HGRE	0.04634942		
2	1	Ld	64	T1c_wholetumor_Energy	0.13456567	-1.68363	0.138059
				T1_wholetumor_GLSZM_GLN	0.08175473		
				T1_wholetumor_GLSZM_LZLGE	-0.13373673		
				T1c_solidcore_GLRLM_GLV	0.04373785		
3	1	Ld	128	T1c_wholetumor_Energy	0.12645875	-1.619012	0.2654483
				T1_wholetumor_GLSZM_LZLGE	-0.11988607		
				T1c_nonenhancing_GLCM_IMC2	-0.06687469		
				T1c_solidcore_GLRLM_HGRE	0.02427534		
4	1	Ld	256	T1c_wholetumor_Energy	0.16796891	-1.716306	0.2128367
				T1_wholetumor_GLCM_IMC1	0.16682933		
				T1_wholetumor_GLSZM_GLV	0.03197865		
				T1c_solidcore_GLSZM_HGZE	0.06016187		
5	1	Eq	32	T1c_wholetumor_Energy	0.20071218	-1.814275	0.3181752
				T1_enhancing_GLSZM_LGZE	-0.07201398		
				T1_solidcore_GLRLM_LRLGE	-0.09984925		
				T1_wholetumor_GLSZM_LZE	-0.07480986		
				T1_wholetumor_GLSZM_GLN	0.01323686		
				T1_wholetumor_GLSZM_GLV	0.09717779		
				Flair_edema_GLSZM_LGZE	-0.13090631		
6	1	Eq	64	T1c_wholetumor_Energy	0.26023195	-1.808891	0.2491751
				T1_necrotic_GLRLM_LRLGE	-0.14304322		
				T1_wholetumor_GLCM_IMC1	0.02059894		
				T1_wholetumor_GLSZM_GLV	0.14805526		
				T1c_nonenhancing_GLCM_IMC2	-0.10845935		
7	1	Eq	128	T1c_wholetumor_Energy	0.17893878	-1.676164	0.1963157
				T1_necrotic_GLRLM_LRLGE	-0.08442391		
				T1_wholetumor_GLCM_IMC1	0.08609644		
				T1_wholetumor_GLSZM_ZSV	0.02661481		
				T1c_nonenhancing_GLCM_IMC2	-0.07298281		
8	1	Eq	256	T1c_wholetumor_Energy	0.19638573	-1.779213	0.1842904
				T1_necrotic_GLSZM_LZLGE	-0.08929799		
				T1_wholetumor_GLCM_IMC1	0.12728308		
				T1c_nonenhancing_GLCM_IMC2	-0.07714256		
9	1	Uf	32	T1c_wholetumor_Energy	0.11009807	-1.612271	0.1671703
				T1_wholetumor_GLCM_IMC1	0.12352298		
				T1c_solidcore_GLRLM_HGRE	0.04856732		
				Flair_nonenhancing_GLSZM_SZLGE	-0.02501661		
10	1	Uf	64	T1c_wholetumor_Energy	0.00226204	-1.400993	0.0466622
				T1_wholetumor_GLSZM_LZLGE	-0.09709271		
11	1	Uf	128	T1c_wholetumor_Energy	0.16931539	-1.694605	0.1051175
				T1_wholetumor_GLCM_IMC1	0.00221989		
				T1_wholetumor_GLSZM_LZLGE	-0.13625046		
				T1c_nonenhancing_GLCM_IMC2	-0.06330023		
				T1c_solidcore_GLRLM_GLV	0.05365544		
				Flair_wholetumor_GLSZM_GLV	0.03938829		
12	1	Uf	256	T1c_wholetumor_Energy	0.187873057	-1.765753	0.2348514
				T1_wholetumor_GLCM_IMC1	0.196553074		
				T1c_solidcore_GLSZM_HGZE	0.067450291		
				Flair_wholetumor_GLSZM_GLV	0.006261932		
13	2	Ld	32	T1_wholetumor_GLSZM_GLN	0.10582461	-1.487693	-0.0619009
				T1_wholetumor_GLSZM_LZLGE	-0.09138091		
14	2	Ld	64	T1_wholetumor_GLSZM_LZE	-0.14722439	-1.663237	-0.0038622
				T1_wholetumor_GLSZM_GLN	0.07147063		
				T2_wholetumor_GLSZM_SZHGE	0.05892811		
15	2	Ld	128	T1_wholetumor_GLCM_IMC1	0.14624762	-1.692613	0.1434881
				T1_wholetumor_GLSZM_GLV	0.05788211		

				T1c_solidcore_GLSZM_SZHGE	0.07332463		
16	2	Ld	256	T1_wholetumor_GLCM_IMC1	0.16614230	-1.529756	-0.0739198
17	2	Eq	32	T1_solidcore_GLSZM_SZE<br>T1_wholetumor_GLCM_DE<br>T1_wholetumor_GLSZM_ZSN	0.02810284<br>0.06280980<br>0.07058818	-1.495821	-0.0293177
18	2	Eq	64	T1_wholetumor_GLCM_IMC1<br>T1_wholetumor_NGTDM_Busyness	0.10908774<br>0.00633358	-1.487193	-0.0376271
19	2	Eq	128	T1_nonenhancing_Kurtosis<br>Flair_edema_Maxvalue<br>T2_nonenhancing_NGTDM_Complexity	-0.01872059<br>0.04697253<br>0.23484965	-1.789826	-0.0653533
20	2	Eq	256	T1_wholetumor_GLCM_IMC1<br>T1_wholetumor_NGTDM_Busyness<br>Flair_nonenhancing_GLCM_SV	0.17961958<br>0.04400307<br>0.09889569	-1.70845	0.2304469
21	2	Uf	32	T1_solidcore_GLSZM_SZE<br>T1_wholetumor_GLCM_IMC1<br>T1_wholetumor_GLSZM_ZSN	0.07344509<br>0.04687577<br>0.12569152	-1.544795	-0.0758735
22	2	Uf	64	T1_wholetumor_GLCM_IMC1<br>T1_wholetumor_GLSZM_GLN<br>T2_wholetumor_GLSZM_SZHGE	0.07955149<br>0.03543966<br>0.01821020	-1.460712	-0.0380348
23	2	Uf	128	T1_wholetumor_GLCM_IMC1<br>T1_wholetumor_GLSZM_GLV<br>T1c_solidcore_GLSZM_SZHGE	0.15085597<br>0.02864715<br>0.04619429	-1.648166	0.1285013
24	2	Uf	256	T1_wholetumor_GLCM_IMC1<br>T1c_solidcore_GLSZM_SZHGE	0.20293481<br>0.08461751	-1.71608	0.2135739
25	3	Ld	32	T1_wholetumor_GLCM_IMC1<br>T1_wholetumor_GLSZM_GLN	0.05291504<br>0.07348525	-1.391169	0.09705524
26	3	Ld	64	T1_wholetumor_GLCM_IMC1<br>T1_wholetumor_GLSZM_ZSN	0.109807322<br>0.003830265	-1.448495	-0.01081814
27	3	Ld	128	T1_wholetumor_GLCM_IMC1	0.1338743	-1.427335	-0.05171302
28	3	Ld	256	T1_wholetumor_GLCM_DE<br>T1_wholetumor_GLCM_IMC1	0.0222978<br>0.1084716	-1.499722	-0.02287799
29	3	Eq	32	T1c_wholetumor_Energy<br>T1_wholetumor_GLCM_Entropy<br>T1_wholetumor_GLSZM_ZSN<br>T1_wholetumor_GLSZM_GLV	0.01077575<br>0.08385488<br>0.04034700<br>0.14960871	-1.677647	-0.1218425
30	3	Eq	64	T1c_wholetumor_Energy<br>T1_wholetumor_GLCM_IMC1<br>T1_wholetumor_GLSZM_GLV<br>T1_wholetumor_GLSZM_ZSV	-0.003670024<br>0.115577276<br>0.119796561<br>0.095959059	-1.660156	0.349093
31	3	Eq	128	T1c_wholetumor_Energy<br>T1_wholetumor_GLCM_IMC1<br>T1c_enhancing_GLCM_MP<br>T2_enhancing_GLRLM_GLV<br>Flair_nonenhancing_GLCM_CP	-0.03077251<br>0.26792819<br>0.08235977<br>-0.01645377<br>0.28517042	-1.891824	0.1531937
32	3	Eq	256	T1_wholetumor_GLCM_IMC1<br>Flair_nonenhancing_GLCM_CP<br>Flair_nonenhancing_NGTDM_Contrast	0.16734563<br>0.11115066<br>0.01557693	-1.590704	0.08607738
33	3	Uf	32	T1c_wholetumor_Energy<br>T1_wholetumor_GLCM_IMC1<br>T1_wholetumor_GLSZM_GLN<br>T1c_nonenhancing_GLSZM_LZLGE	-0.069092683<br>0.085499969<br>0.197654190<br>-0.004657622	-1.745909	-0.1876991
34	3	Uf	64	T1c_wholetumor_Energy<br>T1_enhancing_GLSZM_GLV<br>T1_edema_GLCM_Entropy<br>T1_wholetumor_GLCM_IMC1<br>T1_wholetumor_GLSZM_GLV	-0.09106918<br>0.17714579<br>0.02147564<br>0.14247016<br>0.14396767	-1.911158	0.07763021
35	3	Uf	128	T1c_wholetumor_Energy<br>T2_necrotic_Variance<br>T1_wholetumor_GLCM_IMC1<br>T1_wholetumor_GLSZM_GLV	-0.057636099<br>-0.008142297<br>0.192430202<br>0.133398890	-1.84632	-0.1414833
36	3	Uf	256	T1c_wholetumor_Energy<br>T1_solidcore_GLCM_Energy<br>T1_wholetumor_GLCM_DE<br>T1_wholetumor_GLCM_IMC1<br>T1_wholetumor_GLSZM_GLV	-0.0064466764<br>0.0396974940<br>0.0518395948<br>0.1651373593<br>0.0008508749	-1.685547	-0.02604202

Supplementary Table 5: The prognostic value of all 36 fixed-parameter radiomics signatures. The image standardization parameters, C-Indices, logrank P values and hazard ratios for both training data set and validation data set are listed. VS, QM, GL, Uf, Eq, Ld, HR and CI are short for voxel size, quantization method, gray level, uniform, equal-probability, Lloyd-Max, hazard ratio and confidence interval, respectively.

Index	Parameters			Training Data Set			Validation Data Set		
	VS	QM	GL	C-Index	*P* Value	HR (95% CI)	C-Index	*P* Value	HR (95% CI)
1	1	Ld	32	0.731	<0.001	3.324 (1.628, 6.787)	0.674	0.064	2.347 (0.927, 5.941)
2	1	Ld	64	0.716	<0.001	3.036 (1.523, 6.051)	0.701	0.179	1.944 (0.724, 5.22)
3	1	Ld	128	0.712	<0.001	8.546 (3.498, 20.880)	0.688	0.502	1.510 (0.450, 5.069)
4	1	Ld	256	0.739	<0.001	6.240 (2.689, 14.480)	0.690	0.490	1.379 (0.551, 3.450)
5	1	Eq	32	0.769	<0.001	6.853 (2.931, 16.020)	0.699	0.018	3.585 (1.158, 11.090)
6	1	Eq	64	0.744	<0.001	7.127 (3.118, 16.290)	0.672	0.024	2.673 (1.101, 6.486)
7	1	Eq	128	0.732	<0.001	8.561 (3.727, 19.670)	0.686	0.015	2.778 (1.180, 6.539)
8	1	Eq	256	0.723	<0.001	3.618 (1.823, 7.183)	0.678	0.046	2.394 (0.9885, 5.799)
9	1	Uf	32	0.742	<0.001	5.482 (2.343, 12.832)	0.678	0.576	2.400 (0.892, 6.458)
10	1	Uf	64	0.645	<0.001	6.571 (2.582, 16.724)	0.693	0.010	13.370 (2.876, 62.140)
11	1	Uf	128	0.706	<0.001	4.853 (2.356, 9.997)	0.701	0.008	4.297 (1.879, 9.830)
12	1	Uf	256	0.725	<0.001	5.017 (2.205, 11.421)	0.690	0.064	2.347 (0.927, 5.941)
13	2	Ld	32	0.662	0.001	4.355 (1.683, 11.272)	0.634	0.043	2.683 (0.997, 7.221)
14	2	Ld	64	0.678	0.002	3.402 (1.488, 7.777)	0.611	0.030	2.683 (1.064, 6.764)
15	2	Ld	128	0.697	<0.001	3.484 (1.758, 6.908)	0.672	0.105	1.952 (0.858, 4.441)
16	2	Ld	256	0.640	0.002	3.214 (1.459, 7.081)	0.665	0.031	3.119 (1.054, 9.227)
17	2	Eq	32	0.655	0.003	2.975 (1.410, 6.281)	0.620	0.168	1.798 (0.772, 4.188)
18	2	Eq	64	0.630	0.005	3.062 (1.344, 6.979)	0.647	0.066	2.342 (0.923, 5.938)
19	2	Eq	128	0.662	<0.001	3.223 (1.666, 6.236)	0.543	0.384	1.404 (0.652, 3.025)
20	2	Eq	256	0.670	<0.001	13.600 (4.269, 43.32)	0.595	0.177	1.951 (0.726, 5.242)
21	2	Uf	32	0.664	0.003	3.248 (1.423, 7.410)	0.613	0.080	2.094 (0.901, 4.868)
22	2	Uf	64	0.664	0.002	3.168 (1.450, 6.923)	0.644	0.030	2.683 (1.064, 6.764)
23	2	Uf	128	0.677	0.001	3.065 (1.574, 5.968)	0.663	0.093	1.998 (0.877, 4.549)
24	2	Uf	256	0.688	0.002	3.010 (1.475, 6.145)	0.674	0.255	1.760 (0.657, 4.713)
25	3	Ld	32	0.651	0.002	3.114 (1.465, 6.616)	0.596	0.196	1.778 (0.734, 4.306)
26	3	Ld	64	0.643	0.001	3.422 (1.558, 7.515)	0.678	0.149	1.790 (0.803, 3.989)
27	3	Ld	128	0.648	0.002	3.343 (1.518, 7.347)	0.676	0.006	3.732 (1.379, 10.090)
28	3	Ld	256	0.644	0.005	2.828 (1.338, 5.978)	0.644	0.039	2.369 (1.019, 5.508)
29	3	Eq	32	0.663	0.002	3.206 (1.465, 7.013)	0.626	0.057	2.398 (0.948, 6.608)
30	3	Eq	64	0.670	<0.001	10.07 (3.445, 29.450)	0.632	0.809	1.162 (0.345, 3.907)
31	3	Eq	128	0.721	<0.001	3.862 (1.993, 7.482)	0.593	0.519	1.298 (0.586, 2.876)
32	3	Eq	256	0.685	<0.001	3.217 (1.701, 6.082)	0.663	0.314	1.513 (0.672, 3.407)
33	3	Uf	32	0.681	<0.001	5.360 (2.081, 13.800)	0.603	0.765	1.141 (0.481, 2.707)
34	3	Uf	64	0.704	<0.001	3.162 (1.661, 6.020)	0.684	0.153	1.798 (0.795, 4.067)
35	3	Uf	128	0.677	<0.001	4.608 (1.979, 10.730)	0.634	0.064	2.252 (0.9346, 5.429)
36	3	Uf	256	0.682	<0.001	3.912 (1.811, 8.449)	0.649	0.117	1.881 (0.8439, 4.194)

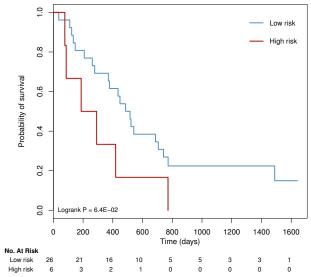

(1) VS=1, QM=Ld, GL=32

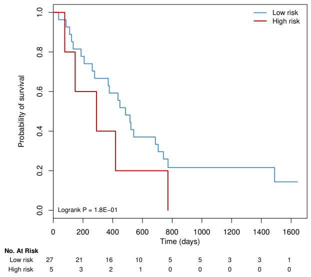

(2) VS=1, QM=Ld, GL=64

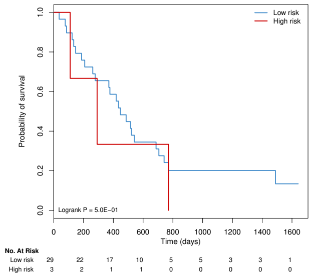

(3) VS=1, QM=Ld, GL=128

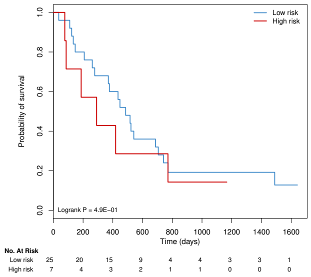

(4) VS=1, QM=Ld, GL=256

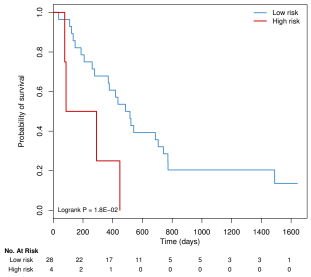

(5) VS=1, QM=Eq, GL=32

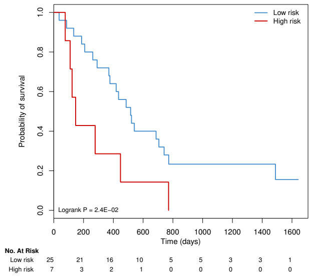

(6) VS=1, QM=Eq, GL=64

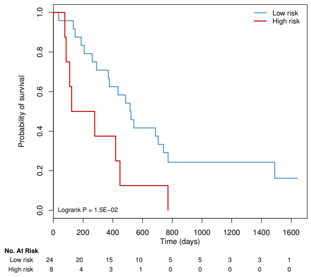

(7) VS=1, QM=Eq, GL=128

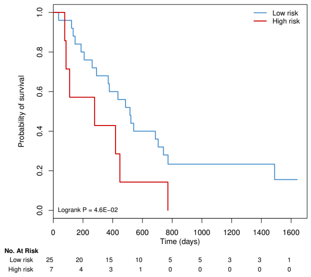

(8) VS=1, QM=Eq, GL=256

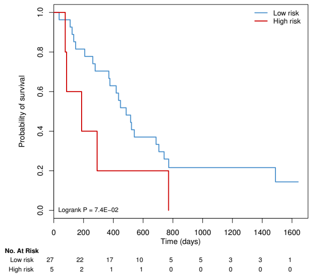

(9) VS=1, QM=Uf, GL=32

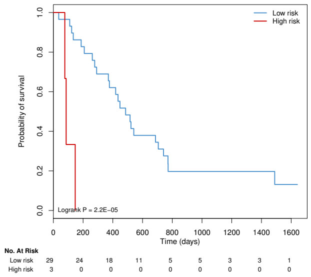

(10) VS=1, QM=Uf, GL=64

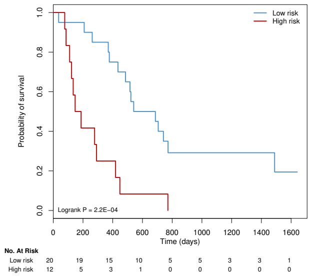

(11) VS=1, QM=Uf, GL=128

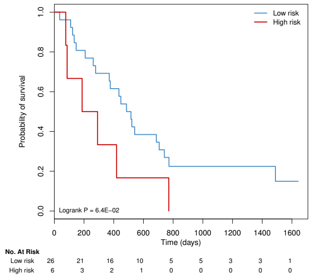

(12) VS=1, QM=Uf, GL=256

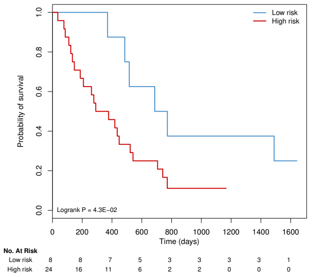

(13) VS=2, QM=Ld, GL=32

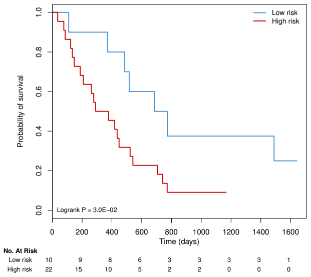

(14) VS=2, QM=Ld, GL=64

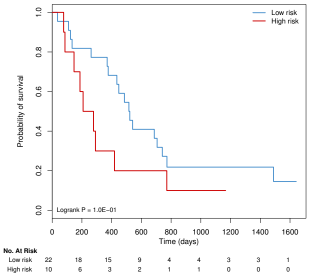

(15) VS=2, QM=Ld, GL=128

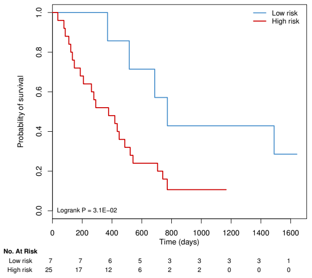

(16) VS=2, QM=Ld, GL=256

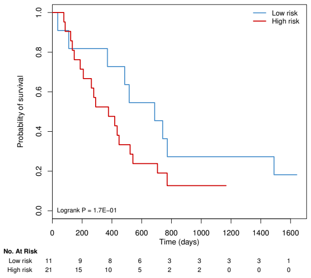

(17) VS=2, QM=Eq, GL=32

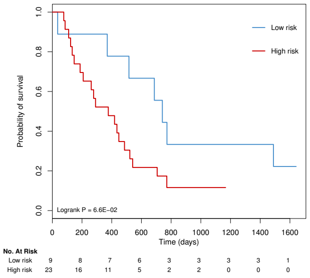

(18) VS=2, QM=Eq, GL=64

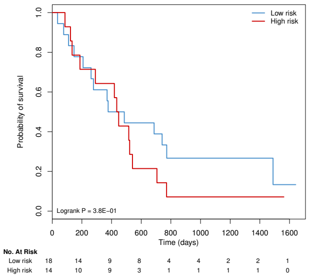

(19) VS=2, QM=Eq, GL=128

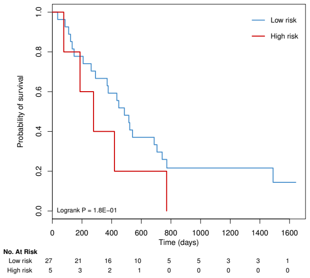

(20) VS=2, QM=Eq, GL=256

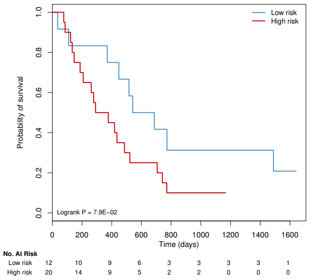

(21) VS=2, QM=Uf, GL=32

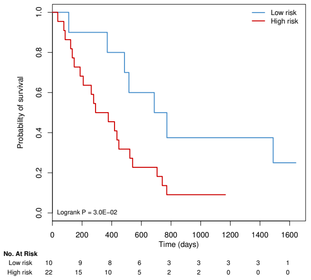

(22) VS=2, QM=Uf, GL=64

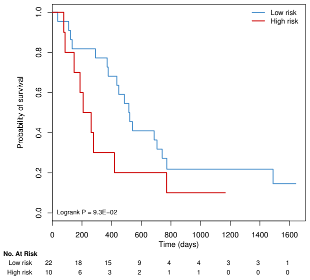

(23) VS=2, QM=Uf, GL=128

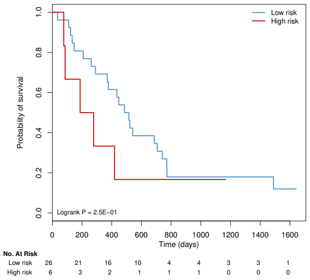

(24) VS=2, QM=Uf, GL=256

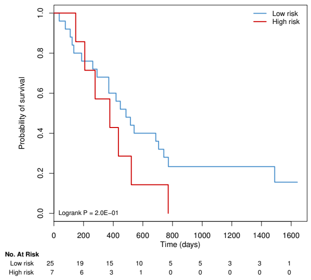

(25) VS=3, QM=Ld, GL=32

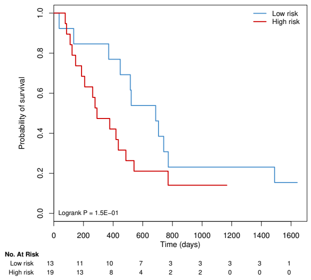

(26) VS=3, QM=Ld, GL=64

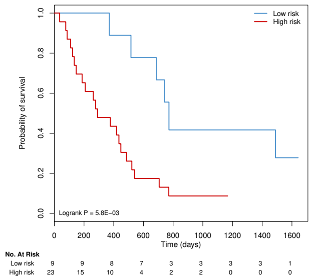

(27) VS=3, QM=Ld, GL=128

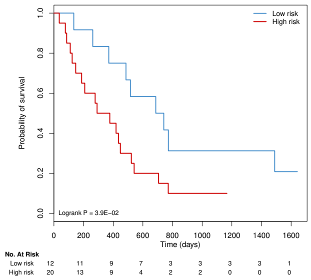

(28) VS=3, QM=Ld, GL=256

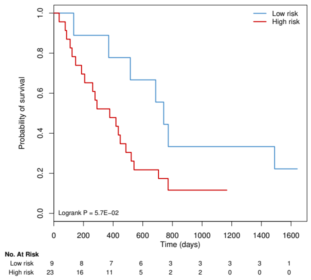

(29) VS=3, QM=Eq, GL=32

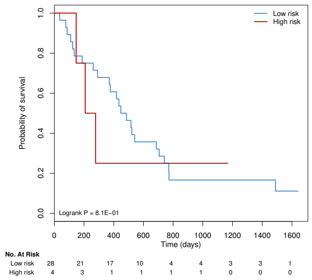

(30) VS=3, QM=Eq, GL=64

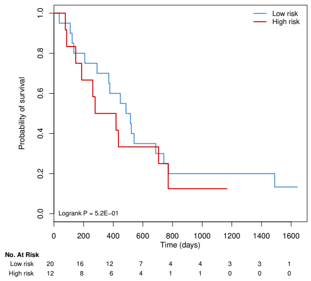

(31) VS=3, QM=Eq, GL=128

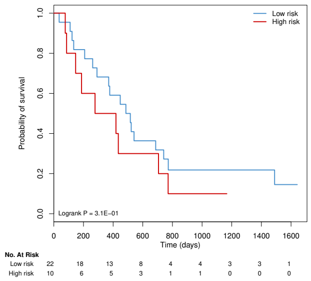

(32) VS=3, QM=Eq, GL=256

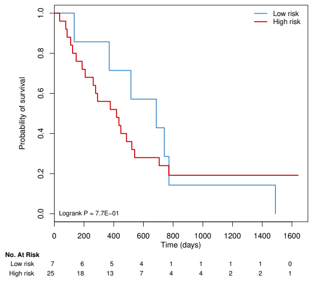

(33) VS=3, QM=Uf, GL=32

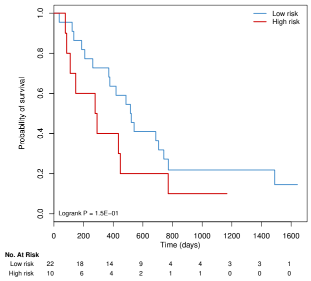

(34) VS=3, QM=Uf, GL=64

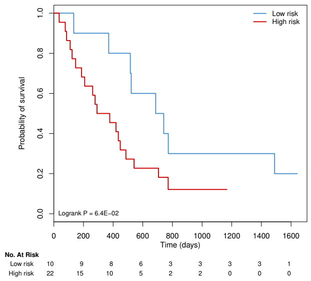

(35) VS=3, QM=Uf, GL=128

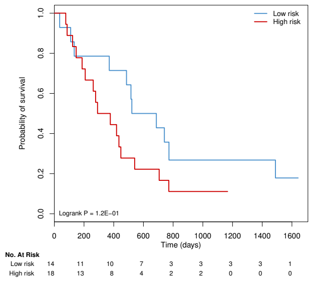

(36) VS=3, QM=Uf, GL=256

Supplementary Figure 2: Kaplan-Meier curves for all 36 fixed-parameter radiomics models with patients in the validation data set. VS, QM, GL, Uf, Eq and Ld are short for voxel size, quantization method, gray level, uniform, equal-probability, and Lloyd-Max, respectively.

References

[1] Mahapatra, D. Analyzing training information from random forests for improved image segmentation. *IEEE Transactions on Image Process.* **23**, 1504–1512 (2014).

[2] Strobl, C., Boulesteix, A.-L., Kneib, T., Augustin, T. & Zeileis, A. Conditional variable importance for random forests. *BMC bioinformatics* **9**, 307 (2008).

[3] Boykov, Y. Y. & Jolly, M.-P. Interactive graph cuts for optimal boundary & region segmentation of objects in nd images. In *Computer Vision, 2001. ICCV 2001. Proceedings. Eighth IEEE International Conference on*, vol. 1, 105–112 (IEEE, 2001).

[4] Boykov, Y., Veksler, O. & Zabih, R. Fast approximate energy minimization via graph cuts. *IEEE Transactions on pattern analysis machine intelligence* **23**, 1222–1239 (2001).

[5] Aerts, H. J. *et al.* Decoding tumour phenotype by noninvasive imaging using a quantitative radiomics approach. *Nat. communications* **5** (2014).
